# Revealing synchrony in pea plants using wavelet coherence analysis

**DOI:** 10.1038/s41598-025-20198-0

**Published:** 2025-10-16

**Authors:** Bianca Bonato, Valentina Simonetti, Umberto Castiello

**Affiliations:** Department of General Psychology, University of Padova, Padova, Italy

**Keywords:** WTC, Wavelet analysis, Time-frequency, Intertwining, Plant synchronization, Pea plant, Computational biology and bioinformatics, Ecology, Plant sciences

## Abstract

In this study, we apply Wavelet Transform Coherence (WTC) analysis—a time–frequency method commonly used in human and animal synchrony research—to examine whether co-potted pea plant dyads exhibit synchronized movement during tendril approach to intertwine. This behaviour allows plants to form braided structures, providing mutual support in the absence of external scaffolds. The results reveal significant coherence, particularly pronounced at the beginning and at the end of the movement sequence. However, this effect varies across dyads, displaying a certain degree of heterogeneity in the coherence patterns. Overall, the analyses indicate that plant movements are temporally structured and non-random; nonetheless, the substantial inter-dyad variability prevents this outcome from being generalized unequivocally. We propose that such synchrony may emerge from embodied mechanisms, including mechanical feedback and chemical signalling. This work extends the application of WTC analysis to plant systems and challenges the assumption that complex coordination requires neural substrates, highlighting the role of distributed, non-neural processes in facilitating cooperative behaviour among living organisms.

**Supplementary Information:**

The online version contains supplementary material available at 10.1038/s41598-025-20198-0.

## Introduction

Climbing plants make use of circumnutation—a rotational movement of plant organs first described by Darwin and Darwin^1^—to explore their surroundings in search of potential supports. This movement can be modulated according to the physical properties of available supports (e.g., thickness)^2^ and the context in which growth occurs (e.g., whether the plant is growing alone or among others)^3,4^. Circumnutation is an inherently dynamic and periodic motion, classified as a rhythmic process^5–7^, and it exhibits the key features of oscillatory behaviour.

In the present study, we investigate circumnutation in *Pisum sativum* (pea plants), paying particular attention to their circumnutative dynamics during dyadic interactions. Specifically, we examine whether two plants, when grown in close proximity without an external support, exhibit synchronized circumnutation patterns that facilitate their mutual approach and culminate in intertwining, a cooperative behaviour wherein tendrils from two individuals interlace to provide shared and structural support^4,8^. This braided configuration will then enhance stability and promotes vertical growth towards the light, in the absence of external supports.

The successful emergence of this behaviour necessitates spatial and temporal coordination, implying a form of motor synchrony between individuals. Such synchronized behaviours are well-documented in animals and humans, often serving as the foundation of coordinated interaction^9,10^.

To study these complex oscillatory dynamics in plants and their potential synchrony underlying circumnutative coordination we employed wavelet coherence (WTC), a powerful method for examining time–frequency relationships between two time series^11^.

WTC identifies localized correlations in both time and frequency domains. This allows for the simultaneous investigation of amplitude and phase relationships, offering a high-resolution view of coordination dynamics^10,12,13^. Wavelet-based methods have demonstrated efficacy in capturing subtle and temporally localized synchrony across diverse domains of life. For instance, beyond human studies, WTC has also been employed to investigate coordination and synchronization patterns in other animals. In rats, WTC has been used to analyse synchrony among optical intrinsic signal imaging time series, evaluating cerebral response differences between stroke-affected and healthy hemispheres^14^. In ecological contexts, WTC has revealed synchronization between lion (*Panthera leo*) activity patterns and moonlight brightness^15^. In another study, it has been shown that movements of African buffalo (*Syncerus caffer*) synchronize when individuals are within approximately 1 km of one another—offering a key spatial benchmark for movement synchrony^15^. Similarly, WTC analysis has been applied to the motor behaviour of insects, particularly *Drosophila melanogaster*, to investigate their high-frequency wing oscillations during flight^16^.

To date, these rhythmic patterns are not limited to organisms with a nervous system, even *aneural* organisms such as jellyfish^17^, slime molds^18^, sponges^19^ and plants^20,21^ exhibit complex oscillatory dynamics.

In the present work, we aim to exploit WTC to study the synchronization of the complex oscillatory dynamics characterizing the circumnutation of two pea plants when acting in a dyad to perform the intertwining. We hypothesize that if to accomplish the intertwining behaviour pea plant dyads requires temporal synchronization, then this will be detectable through WTC analysis. For the sake of the present study, we interpret the term *synchronization* in its broadest and most inclusive sense, to indicate coupling without a specific fixed phase shift connotation, as conceptualized by Pikovsky and colleagues^22^. This definition allows for a degree of variability in phase relations while still capturing the essence of coordinated rhythmic behaviour.

## Materials and methods

### Subjects

Sixteen *Pisum sativum* L. var. *Saccharatum* plants (Fratelli Ingegnoli S.p.a., Milan, Italy) were selected for this study (see Table 1). The var. *Saccharatum* was confirmed by formal identification provided by IGA Technology Services S.r.l (Udine, Italy). The plants were arranged in pairs, forming eight dyads.

**Table 1:** Sample description.

Sample	16 plants, arranged in 8 dyads
Germination	6 days
Distance from the other plant	10 cm
Time required to intertwine	Mean = 18 days, *sd = 5.54 days*
Duration of the analysed movement (from movement onset)	Real- dyads: Mean = 1676.63 min, *sd = 397.33 min* Pseudo-dyads: Mean = 1427.63 min, *sd = 326.27 min*
Plant height at the end of experiment (from origin to apex)	All: Mean = 206.38 mm, sd = 63.37 mm Handlers: Mean = 218 mm, sd = 60.46 mm Graspers: Mean = 194.75 mm, sd = 64.07 mm

### Germination and growth conditions

The seeds were germinated for 5 days in filter paper strip soaked with water at 1.5 cm from each other and 0.5 cm from the top of the strip. Seed orientation showed the hilum and micropyle oriented downward. Then healthy seedlings of the same developmental stage were selected and planted in a plastic pot. The pot used was 30 cm in diameter and 14 cm in height. The pot used for the social condition was 30 cm in diameter and 14 cm in height. The pots were filled with silica sand (type 16SS, dimension 0.8/1.2 mm, weight 1.4 t/m³). Silica sand offers superior drainage and aeration due to its coarse, uniform particle size, which helps prevent waterlogging and promotes healthy root development. Additionally, it is chemically inert and sterile, minimizing variability due to microbial activity. This inert quality also allows for precise control of nutrient and water delivery.

**Fig. 1:**
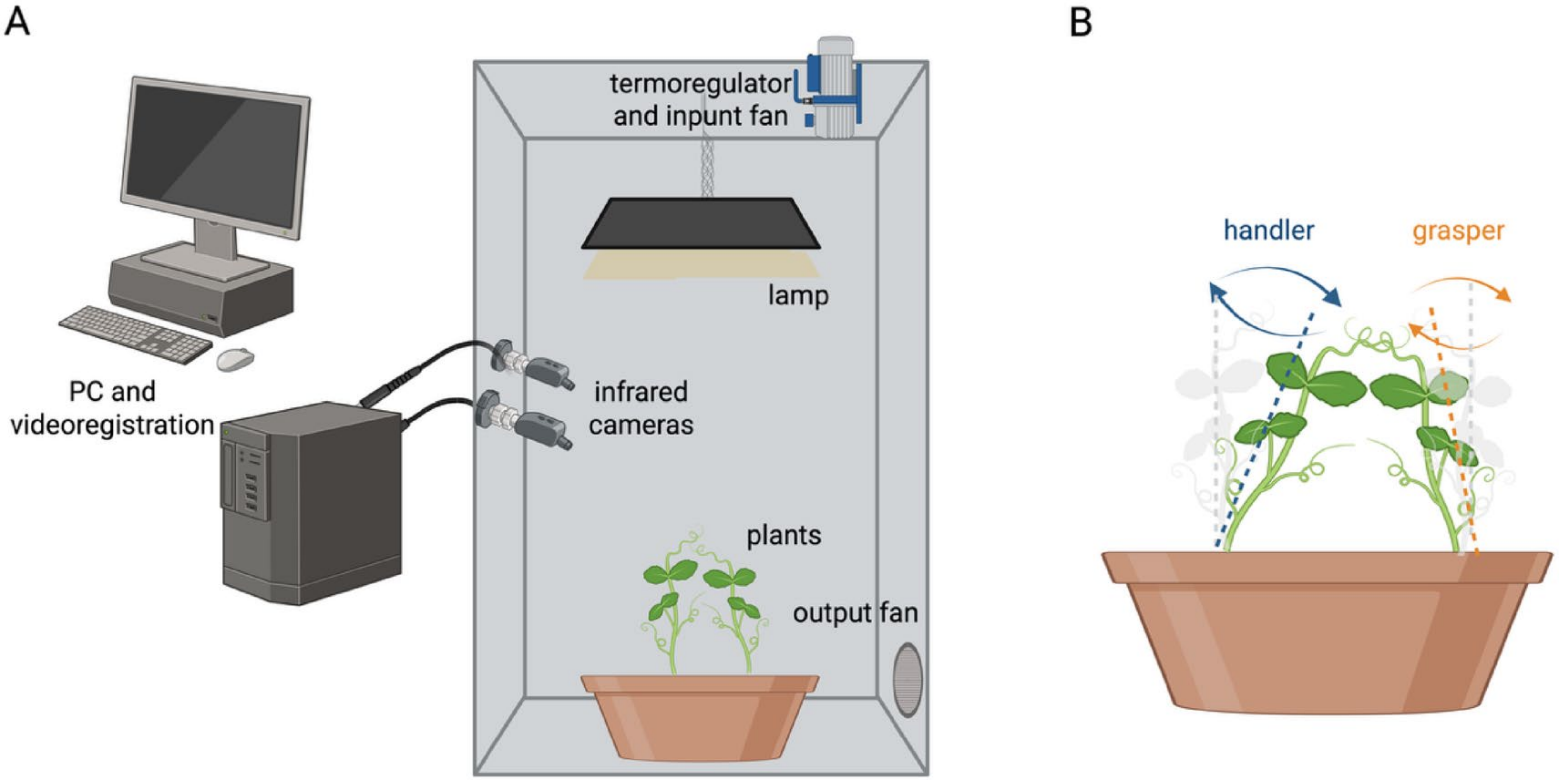
Experimental setup. Panel (**A**) represents the growth chamber with all elements and instruments; Panel (**B**) represents the interaction of the two plants for the “real” dyads. Plants move one towards the other as they accomplish the intertwining behaviour with their tendrils. As represented by the blue lines, the “handler” plant bend towards the other; the “grasper” plant represented by orange lines, remain fix along its own axis, finalizing the grasping phase. This complementary behaviour allows the two plants forming horizontal braided structures that stabilize the dyad and provide mutual support to grow vertically.

At the beginning of each treatment, the pots for the individual and social condition were watered and fertilized using a half-strength solution culture (Murashige and Skoog Basal Salt Micronutrient Solution; 10x, liquid, plant cell culture tested; SIGMA Life Science). The soil volume and the solution culture allowed for adequate soil and fertilizing conditions for the plants in both the individual and the social condition. The plants were watered 3 times a week. Each pot was enclosed in a growth chamber (Cultibox SG combi 80 × 80 × 160 cm) so that the plants could grow in controlled environmental conditions (see Fig. 1). The chamber air temperature was set at 26 °C and remained constant between 24 °C and 26 °C during the day–night cycle; the extractor fan was equipped with a thermo-regulator (TT125; 125 mm-diameter; max280 MC/H vents) and there was an input-ventilation fan (Blauberg Tubo 100–102 m3/h). The two-fan combination allowed for a steady air flow rate into the growth chamber with a mean air residence time of 60 s. The fan was placed so that air movement did not affect the plants’ movements. Plants were grown with an 11.25 h photoperiod (5.45 am to 5 pm) under a cool white LED lamp (V-TAC innovative LED lighting, VT-911–100 W, Des Moines, IA, USA or 100 W Samsung UFO 145 L m/W—LIFUD) that was positioned 57 cm above each seedling. Photosynthetic Photon Flux Density at 57 cm under the lamp in correspondence with the seedling was 350 µmolph/m2s (quantum sensor LI-190R, Lincoln, Nebraska USA). Reflective Mylar^®^ film of chamber walls allowed for better uniformity in light distribution.

### Experimental conditions

To study synchronization patterns in our pea plants, we capitalized on a paradigm that was successful in identifying motor consonance in pea plants via correlational analysis^4^. We considered two experimental conditions: (i) an intertwining condition in which two plants were potted together with no potential supports in the surrounding (i.e., real dyad condition). In this context the plants assume complementary roles, which are characterized by specific kinematic features functional to coordinate the intertwining of their tendrils to mutually support each other^4^ (see Fig. 1). The *handler* plant exhibits a pronounced leaning behaviour toward the other plant to initiate the intertwining process. The *grasper* plant coils its tendrils around the handler^4^; (ii) a condition in which we took data of plants from different dyads (i.e., *grasper* plant from a dyad; *handler* plant from another dyad) and randomly paired to create eight artificial shuffled dyads termed as “pseudo dyad”.

### Kinematic data collection

For each growth chamber, a pair of RGB-infrared cameras (i.e. IP 2.1 Mpx outdoor varifocal IR 1080P) were placed 110 cm above the ground, spaced at 45 cm to record stereo images of the plant. The cameras were connected via Ethernet cables to a 10-port wireless router (i.e. D-link Dsr-250n) connected via Wi-Fi to a PC and the frame acquisition and saving process were controlled by CamRecorder software (Ab.Acus s.r.l., Milan, Italy). To maximize the contrast between the peas’ anatomical landmark (i.e., the tendrils) and the background, black felt velvet was fixed on some sectors of the boxes’ walls. The intrinsic, extrinsic, and lens distortion parameters of each camera were estimated using a Matlab Camera Calibrator App. Depth extraction from the single images was conducted by taking 20 pictures of a chessboard (squares’ side 18 mm, 10 columns, 7 rows) from multiple angles and distances in natural non-direct light conditions. For stereo calibration, the same chessboard used for the single-camera calibration process was placed in the middle of the growth chamber. The two cameras took simultaneous pictures used to extract the stereo calibration parameters. In accordance with the experimental protocol, the camera synchronously acquired a frame every 3 min (frequency 0.0056 Hz). The experiment started with the positioning of the selected plants in a pot inside the growth chambers. At that time the cameras started acquiring. For the analysis, we selected the window in which the circumnutation sectors of interest occurred. To elaborate, the plants develop several sets of branches from specific points of the stem, which we refer to as *nodes*. For both the handler and the grasper, we analysed the last branch, focusing on the tendril emerging from it. Pea plants can variably produce up to three tendrils and we chose to analyse the one that touched the other plant first. The initial frame was defined as the frame in which the considered leaf’s tendrils were visible from the apex. The end of the plant movement was defined as the frame at which the tendrils of one plant coiled around the other plant. That is, when the intertwining started. An ad hoc software (SPROUT, Ab.acus s.r.l., Milan, Italy)^23^ developed in Matlab and Python was used to identify anatomical points on the pictures taken by the two cameras and to track their position frame by frame. 2D trajectories obtained from each camera together with calibration parameters were used to reconstruct the 3D trajectory of each point. The anatomical landmark of interest, namely the tip of the tendril, were identified post hoc. No physical marker was positioned on the plant. The tracking procedures were at first performed automatically throughout the time course of the movement sequence using the Kanade-Lucas-Tomasi algorithm on the frames each camera acquired after distortion removal. The tracking was manually verified by the experimenter, who checked the position of the markers frame by frame.

### Dependent measure

The dependent variable used to test our experimental hypothesis was the mean of the magnitude-squared WTC of the tendril position along the x and z axes). This variable was adopted as a measure of the correlation between the movements of the two plants in the time-frequency domain.

### Wavelet coherence (WTC) analysis

**Fig. 2:**
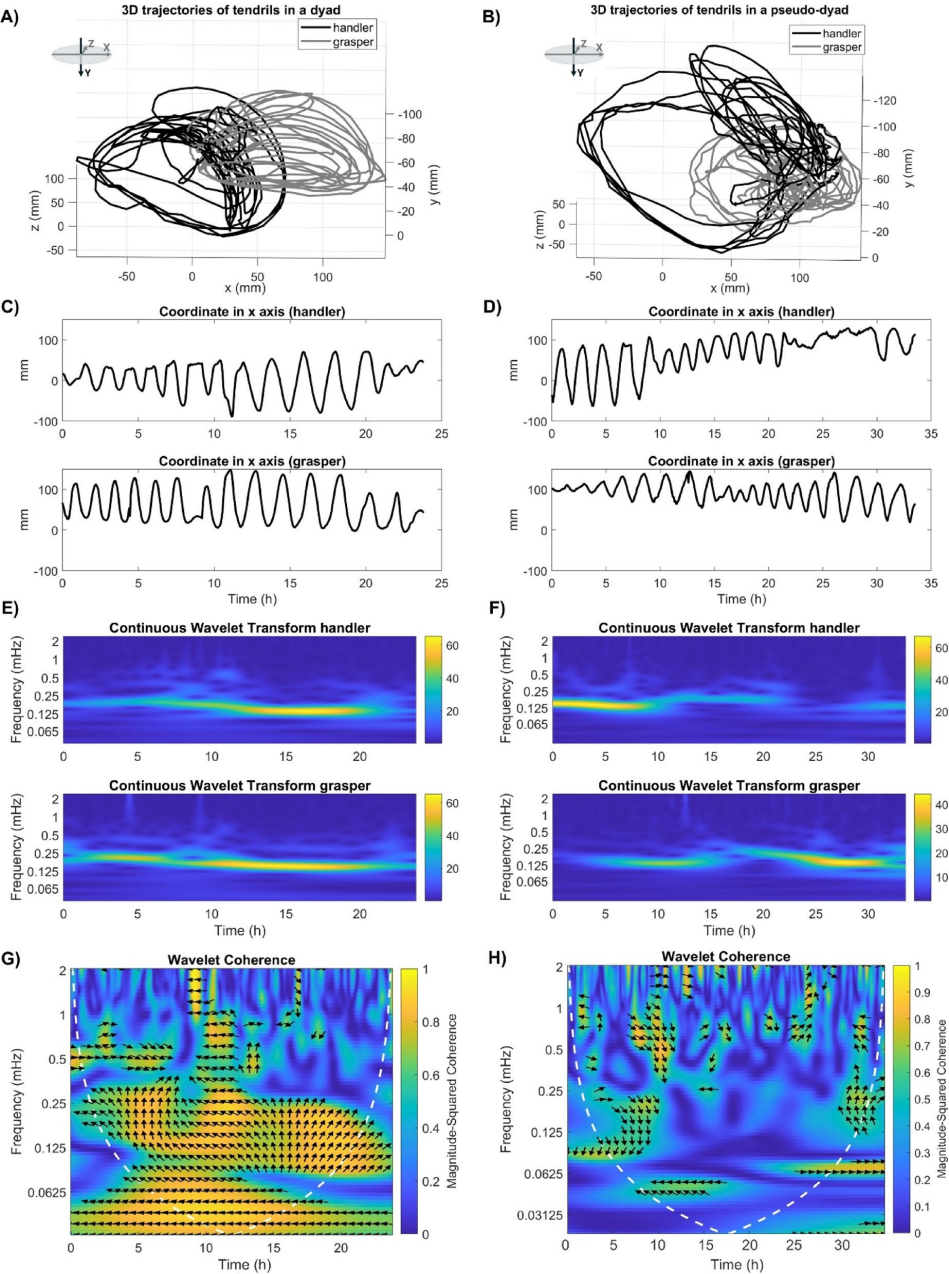
(**A**) 3D trajectories of the tendril of both handler and grasper for a dyad showing high coherence. Y axis represent the vertical axis while X and Z axes are on the horizontal plane; **B**) 3D trajectories of the tendril of both handler and grasper for a pseudo-dyad showing lower coherence. Y axis represent the vertical axis while X and Z axes are on the horizontal plane; **C**) Time series of the x coordinate for the tendrils of the handler and the grasper plant corresponding to the 3D trajectory in A); **D**) Time series of the x coordinate for the tendrils of the handler and the grasper plant corresponding to the 3D trajectory in **B**); **E**) continuous wavelet transform (CWT) of the time series of x coordinate presented in **C**) corresponding to a real dyad with high final coherence; **F**) continuous wavelet transform (CWT) of the time series of x coordinate presented in **D**) corresponding to a pseudo-dyad with lower final coherence; **G**) WTC matrix obtained from the CWTs in **E**); **H**) WTC matrix obtained from the CWTs in F). In the WTC matrices, the arrows represent the lead–lag phase relationship between the two time series at each time–frequency point. A zero-phase difference, represented by arrows pointing directly to the right, indicates that the series are perfectly in phase at that scale. Conversely, arrows pointing to the left indicate anti-phase (i.e., the series move in opposite directions). Arrows pointing right-down or left-up indicate that the first variable (handler) is leading, while arrows pointing right-up or left-down suggest that the second variable (grasper) is leading. Phase arrows are only displayed in regions where the squared wavelet coherence exceeds the threshold of 0.5. The white dotted lines in WTC matrices indicate the cone of influence (COI), where edge effects become significant. Interpretation outside the COI is less reliable due to boundary artifacts.

We applied an approach similar to that used for the analysis of human movements^12,24^. We evaluated the movement synchrony in the time–frequency domain using WTC, that can be considered as the local correlation between two Continuous Wavelet Transforms (CWTs)^11^. For this study, we started from the 3D trajectory of the tendrils and we extracted the time series of the position in the x, y and z axes (in millimetres) over time (in seconds), where the y axis represents the vertical dimension, and the x and z axes lie in the horizontal plane (see Fig. 2; see Figures S1−7 in Supplementary material for additional examples of real-dyads). Since tendril movements during nutation evolve mainly in the horizontal plane, we expected x and z axes to be mainly involved in the oscillatory movement. The time series obtained from the real dyads are temporally aligned and have the same duration because they have been acquired simultaneously and are expression of the same joint action of intertwining. To correctly compare the time series of the pseudo-dyads, we temporally aligned them and adjusted for differences in the duration of the intertwining movement. Specifically, for each pseudo-dyad, we aligned the time series of the two plants starting from their respective movement onsets - defined as the frame in which the tendrils of the observed leaf first became visible from the apex - up to the shorter of the two movement durations.

The WTC of the collected time series in the x and z axes for all dyads was calculated using MATLAB (MathWorks, version 2024b) Wavelet Toolbox^11^. The WTC was computed using the analytic Morlet wavelet. In line with previous studies, we visualized coherence here using colours ranging from blue (no synchrony) to warmer colours (perfect synchrony^24,25^). Figure 2 summarizes all the steps taken for the WTC analysis for two sample dyads (a real one and a pseudo one). The figure shows an example of the extraction of the time series of the position in the x and z plane (Fig. 2C and D) and the calculation of CWTs (Fig. 2E and F) and WTC matrices (Fig. 2G and H).

### Data analysis

To quantify the overall level of synchrony between tendrils’ movements in a dyad, we computed the mean of the magnitude-squared WTC of the tendril position in the x and z axes of the 3D movement. The mean of the WTC for each plant was calculated by averaging all elements of the WTC matrix obtained (as in^24^. To assess the differences between the population for real dyads and the population of pseudo dyads, we employed a non-parametric Mann-Whitney U test. We also calculated the effect size using Cliff’s delta.

## Results

**Fig. 3:**
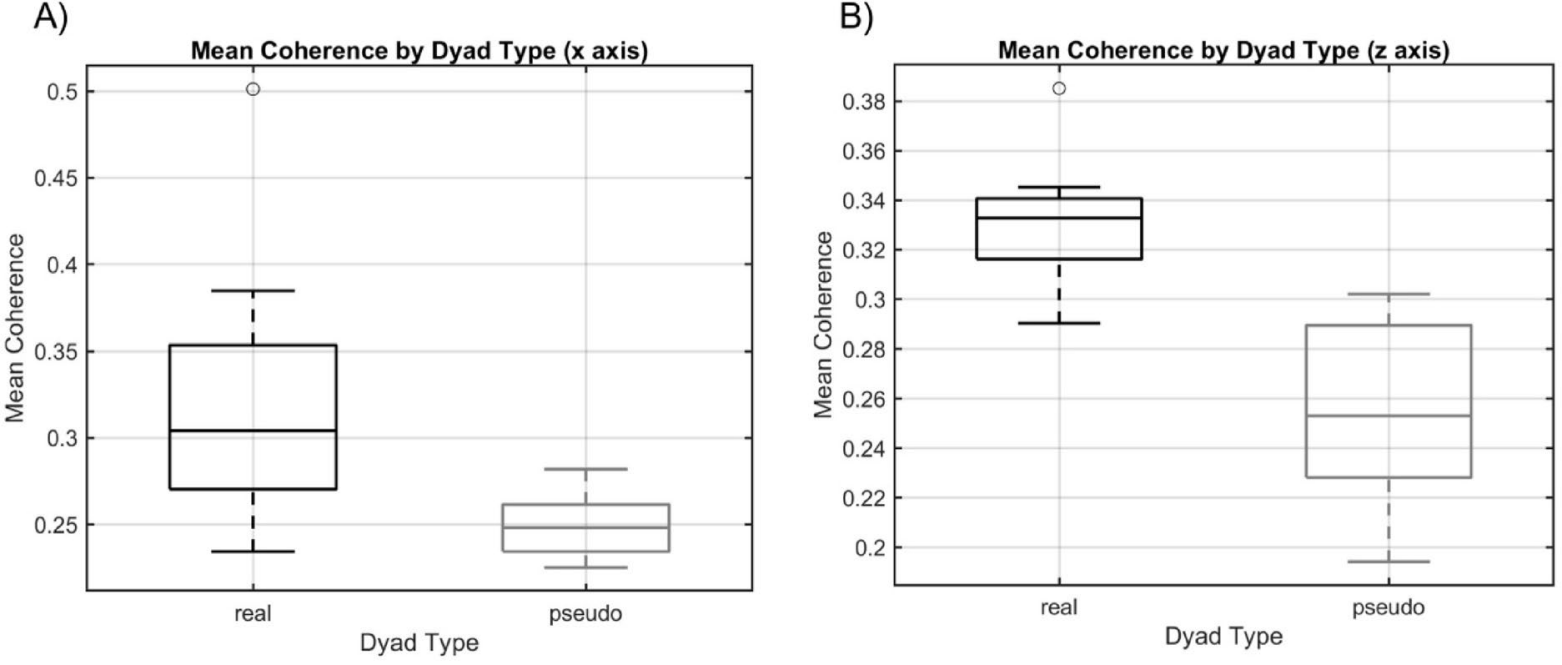
show the boxplots of the distribution of the mean WTC values for each dyad type both for the x axis (Fig. 3A) and for the z axis (Fig. 3B).

Figure 3. (**A**) Mean coherence for pseudo- dyads (*n* = 8) and real dyads (*n* = 8) calculated as the mean of the magnitude-squared WTC of the tendril position on the x axis, *p* <.05. **B**) Mean coherence for pseudo-dyads (*n* = 8) and real (*n* = 8) dyads calculated as the mean of the magnitude-squared WTC of the tendril position on the z axis, *p* <.05.

Results of the Mann-Whitney U test showed that the mean WTC for real dyads was significantly higher than for pseudo dyads with a moderate to large effect for both the movement along the x axis (*p*-value = 0.014763, Cliff’s Delta = 0.71875) and along the z axis (*p*-value = 0.028127, Cliff’s Delta = 0.65625). This suggests that pea plants synchronize their movement during intertwining when part of real dyad and that this synchronization is functional to the correct execution of the joint action and not only to the growth phase.

**Fig. 4:**
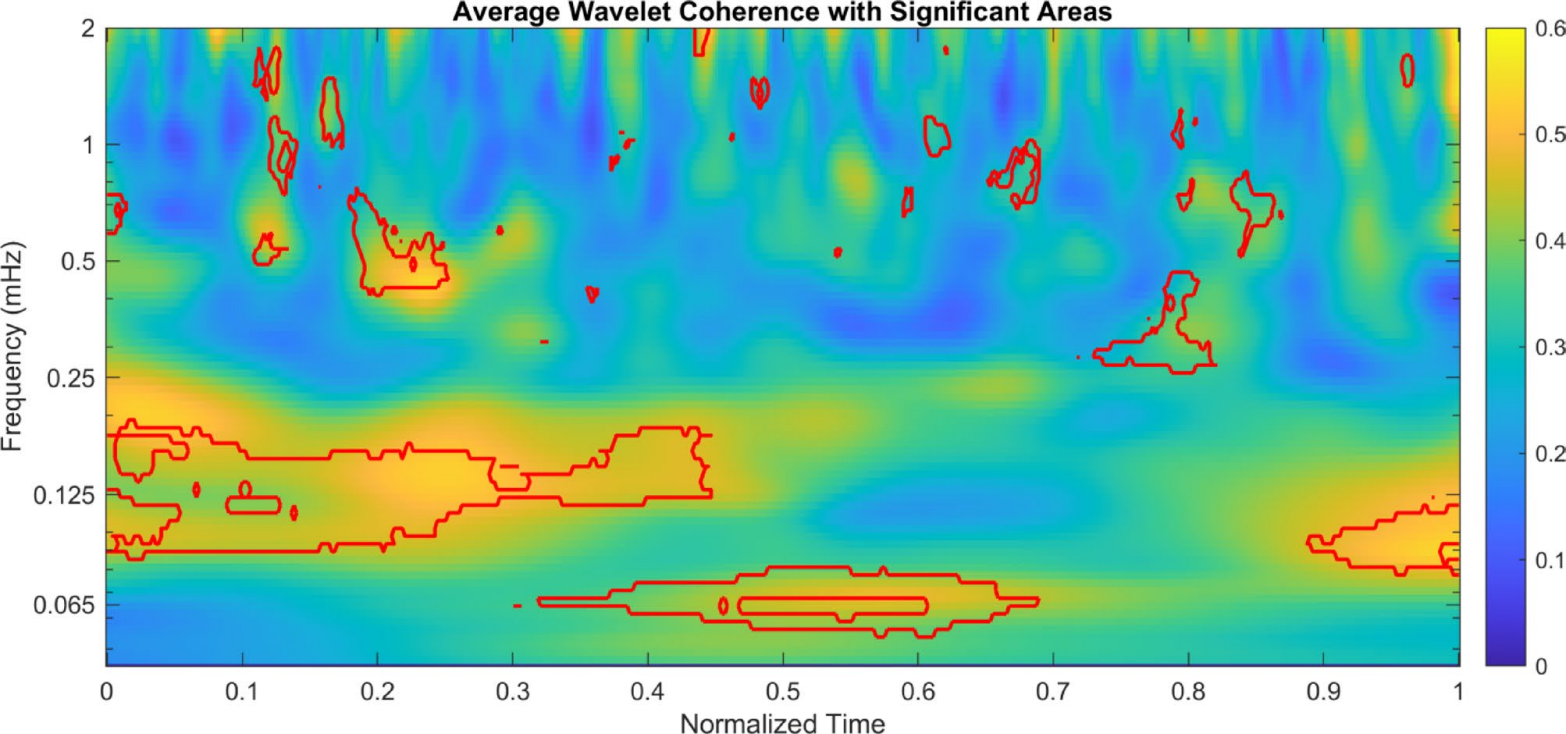
Average WTC matrix obtained from the average of the WTC matrices extracted from all the real dyads across the x direction. To perform the average, we normalized movement time to make it comparable across dyads. Areas outlined in red indicate regions where the difference between real and pseudo dyads is statistically significant (q < 0.05, FDR-corrected), based on a directional (one-tailed) pixel-wise Mann–Whitney U test testing whether real dyads values > pseudo dyads values.

To identify frequency bands and time intervals showing high coherence and to provide a general temporal structure of coordination to reach intertwining, we computed an average WTC matrix as the average for all the matrices obtained for the real dyads after the normalization of movement time. We also run a pixel-wise Mann-Whitney U test to compare the areas in the map between real and pseudo dyads. Figure 4 shows the obtained average WTC matrix where the areas where the difference between real dyads and pseudo dyads is significant (*p* <.05) are enclosed inside red lines.

## Discussion

In the present study, we focused on the oscillatory circumnutation movement of pea plants, which is by nature rhythmic and directional, for evaluating dynamic relational behaviours through WTC analysis. Our results demonstrate significant time- and frequency specific synchronization between individuals in real dyads, indicating a form of complementary behavioural coordination. This coordination appears to facilitate goal-directed movement, suggesting that both agents temporally align their behaviours to optimize the progressive approach to achieve the intertwining.

WTC analysis, which has been extensively validated in human behavioural studies, provides a robust framework for assessing these joint activities. In human research, increased coherence in movement has been consistently observed when dyads engage in coordinated, complementary tasks, as opposed to pseudo-dyads in which individual behavioural trajectories are randomly paired^24^. Analogously, our plant data reveal significant higher coherence in real dyads with respect to pseudo-dyads, the latter constructed by virtually random pairing time-series from different individuals. This distinction reinforces the interpretation that the observed synchrony in real dyads is not attributable to stochastic alignment, but rather to genuine interactive dynamics. Our idea is that the real dyad acts as a coordinated system to reach a common goal while the pseudo-dyad, virtually generated and not interacting in real space, does not act as a coordinated system. In this way we could prove that the dynamics of the plant are not driven only by the fact that they grew together with another plant, but that those dynamics are dependent on the specific interactions that the two plants mutually experienced along their growth.

In particular, WTC analysis of the progressive approach-to-intertwine process comparing real dyads and pseudo-dyads revealed distinct, temporally localized areas of high coherence, particularly at the initial and terminal segments of the normalized movement trajectory (Fig. 4) for the real-dyads. Specifically, on average, coherence was higher during the early 0–40% and the final 90–100% of the movement cycle, whereas a reduction in coherence was observed during the intermediate phase (50–90%). A closer inspection of this patterning outlines a region of significance around 40% and 60% of movement duration of approximately 0.065mHz, a lower frequency with respect to that detected for the initial and final regions (∼0.125 mHz). This temporal differentiation suggests the presence of functionally distinct phases in the coordination process in the real dyads with respect to the pseudo-dyads: an initial phase of mutual orientation and a terminal phase characterized by synchronization to achieve task completion. In general terms, this pattern could align with some findings from human coordination research^26–28^.

A point worth noting, is that the observed pattern is not consistent across real dyads (see Figures S1−7 in Supplementary material for depiction of all real-dyads). The result shown in Fig. 4 is driven by variability across dyads, with some exhibiting high coherence at the beginning, others at the end of the movement. Specifically, one dyad shows high coherence during the entire movement (Fig. 2G). Three dyads exhibited high coherence during the first phase of the movement (see Figures S1, S3, and S4 in the supplementary material), while for three dyads high coherence was evident toward the end of the movement (see Figures S5, S6, and S7 in the supplementary material). In addition, coherence was also observed during the intermediate phase (see Figure S2 in the supplementary material). We interpret these findings in light of the inherent variability characteristic of motor control in general, and of complementary actions in particular^22^. Taken together, the analyses suggest that plant movements are temporally structured and not random; however, the marked inter-dyad variability limits the extent to which the pattern showed in Fig. 4 can be generalized. We suggest, with a certain degree of caution, that this form of dynamic coordination, is an organism independent principle subtending complementary behavior. Having said that, our intention is not to draw a direct comparison between humans and plants, but rather to highlight certain mechanisms that may reflect, in some way, similar patterns of coordination observed across taxa.

This cross-species convergence in coordination processes suggests the presence of a fundamental mechanism of synchrony that may be invariant across biological taxa. Crucially, unlike humans, plants lack a central nervous system or mirror neuron structures. This imply that this synchronization emerges from alternative biophysical or biochemical mechanisms.

At this stage the central question is how such coordination can emerge in the absence of a nervous system^4,29–31^. Drawing from existing literature, we propose that plant coordination may be mediated through growth-driven movement modulated by mechanical cues—such as touch, tension, and pressure (for a review see^32^—as well as chemical signalling, such as volatile organic compounds (VOCs^33^;. These mechanisms may enable plants to detect and respond to each other’s presence, thereby facilitating dynamic adjustment of growth patterns in a coordinated manner. In the present study, plants could perceive each other through the root system, touching their roots but also exchanging roots exudates in the rhizosphere that allows to detect neighbors and recognize their identity^34^. Further, another potential mechanism that is worth mentioning and that could be relevant in our case, involves electromagnetic cues such as light reflection^35,36^. This may provide plants with primitive yet meaningful light information of their surroundings.

Further exploration of how plants modulate growth in response to environmental and inter-individual cues may yield critical insights into the foundational principles of collective behaviour in biological systems.

Future studies should investigate other aspects related to synchronization in plant movements and how this investigation should be carried out. In our vision, each plant inside a dyad is a self-sustained oscillator capable of generating its own rhythm and this rhythm is adjusted due to weak interaction with the other plant of the dyad. According to Pikovsky et al.^22^, the study of synchronization should include the evaluation of the adjustment of rhythms in a certain range of systems’ mismatch. If the frequency of one oscillator is slowly varied, the second system should follow this variation. This could be achieved by introducing a form of perturbation or the modulation of one plant’s rhythm while monitoring the response of the other to observe rhythm re-adjustment. This could be achieved with temporary mechanical constraint of one of the two plants’ movement (e.g., tying the circumnutating branch for the time of few nutations) and subsequent removal of the constraint. In addition, it would be interesting to evaluate coupling strength in plant dyads by varying the distance between plants.

So far, our findings challenge the assumption that complex coordination necessitates neural substrates, and instead support the view that embodied, distributed mechanisms—grounded in physical interaction and environmental feedback—can give rise to sophisticated forms of cooperation.

The key innovation of this study lies in the application of a time-frequency based analytical framework to plant behaviour. By leveraging on WTC, we open new avenues for exploring biological oscillatory patterns across diverse life forms, providing a strong methodological approach to studying the dynamic interaction behind processes that underpin life on Earth.

## Supplementary Information

Below is the link to the electronic supplementary material.

Supplementary Material 1

## Data Availability

The datasets generated during and/or analysed during the current study are available in the zenodo repository, at this link https://doi.org/10.5281/zenodo.15606745. The raw data shared with Bonato et al., (2024) are available at this link https://zenodo.org/records/10400719.
