# Supplementary material for: Revealing synchrony in pea plants using wavelet coherence analysis
Source: Sci Rep. 2025 Oct 16;15:36226. doi: 10.1038/s41598-025-20198-0 (PMC12533233; doi:10.1038/s41598-025-20198-0)
Supplement: Supplementary file 1 — Supplementary Material 1 [file 41598_2025_20198_MOESM1_ESM.pdf]

Additional sample real dyad #1

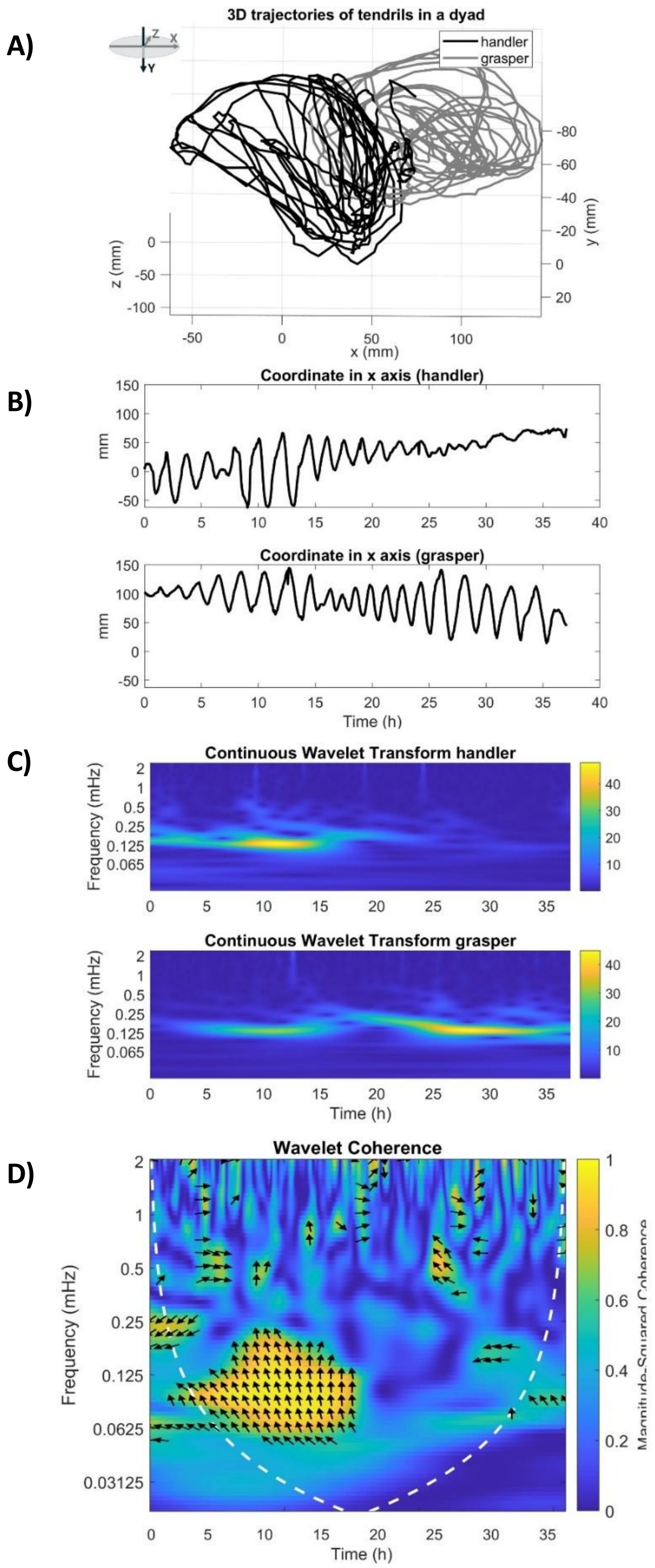

Figure S1. A) 3D trajectories of the tendril of both handler and grasper for a sample real dyad. Y axis represent the vertical axis while X and Z axes are on the horizontal plane; B) Time series of the x coordinate for the tendrils of the handler and the grasper plant corresponding to the 3D trajectory in A); C) continuous wavelet transform (CWT) of the time series of x coordinate presented in B); D) WTC matrix obtained from the CWTs in C). In the WTC matrix, the arrows represent the lead-lag phase relationship between the two time series at each time-frequency point. A zero-phase difference, represented by arrows pointing directly to the right, indicates that the series are perfectly in phase at that scale. Conversely, arrows pointing to the left indicate anti-phase (i.e., the series move in opposite directions). Arrows pointing right-down or left-up indicate that the first variable (handler) is leading, while arrows pointing right-up or left-down suggest that the second variable (grasper) is leading. Phase arrows are only displayed in regions where the squared wavelet coherence exceeds the threshold of 0.5. The white dotted lines in WTC matrices indicate the cone of inffuence (COI), where edge effects become significant. Interpretation outside the COI is less reliable due to boundary artifacts.

Additional sample real dyad #2

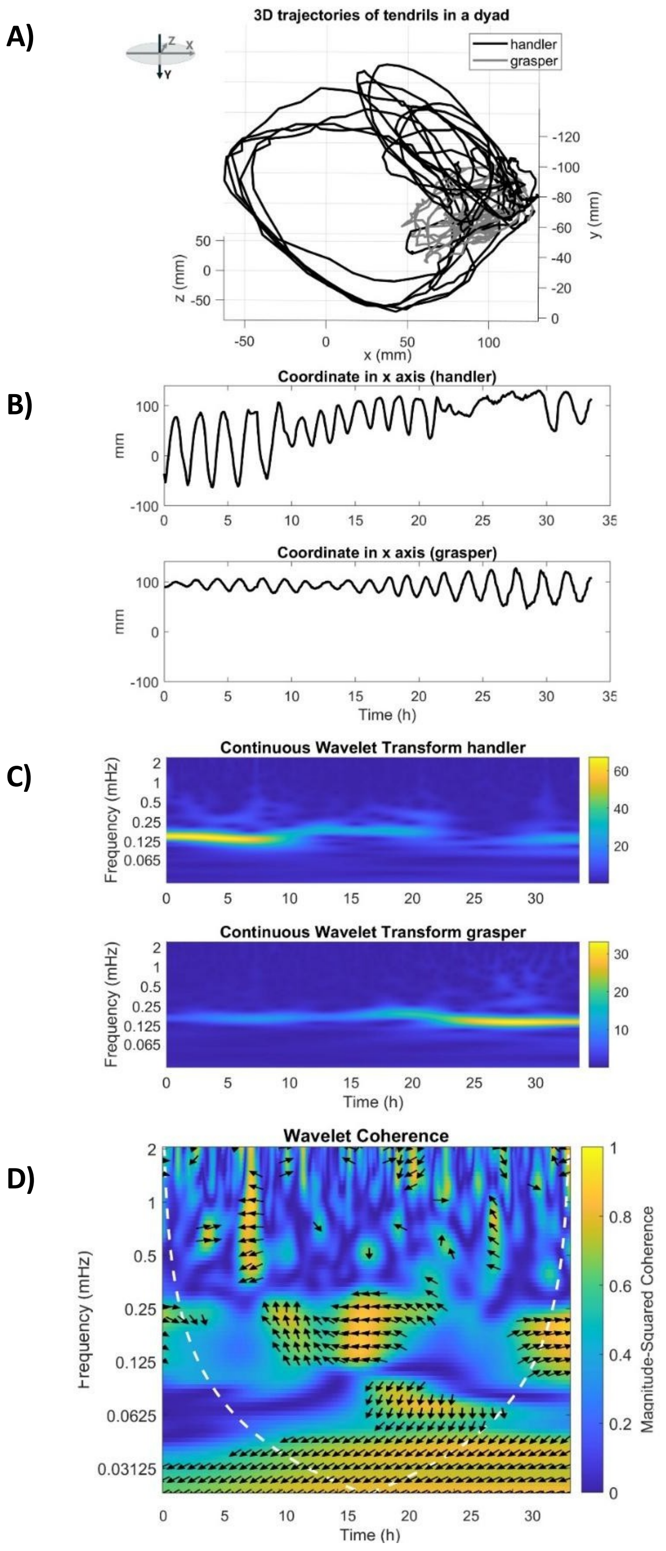

Figure S2. A) 3D trajectories of the tendril of both handler and grasper for a sample real dyad. Y axis represent the vertical axis while X and Z axes are on the horizontal plane; B) Time series of the x coordinate for the tendrils of the handler and the grasper plant corresponding to the 3D trajectory in A); C) continuous wavelet transform (CWT) of the time series of x coordinate presented in B); D) WTC matrix obtained from the CWTs in C). In the WTC matrix, the arrows represent the lead-lag phase relationship between the two time series at each time-frequency point. A zero-phase difference, represented by arrows pointing directly to the right, indicates that the series are perfectly in phase at that scale. Conversely, arrows pointing to the left indicate anti-phase (i.e., the series move in opposite directions). Arrows pointing right-down or left-up indicate that the first variable (handler) is leading, while arrows pointing right-up or left-down suggest that the second variable (grasper) is leading. Phase arrows are only displayed in regions where the squared wavelet coherence exceeds the threshold of 0.5. The white dotted lines in WTC matrices indicate the cone of inffuence (COI), where edge effects become significant. Interpretation outside the COI is less reliable due to boundary artifacts.

Additional sample real dyad #3

A)

B)

C)

D)

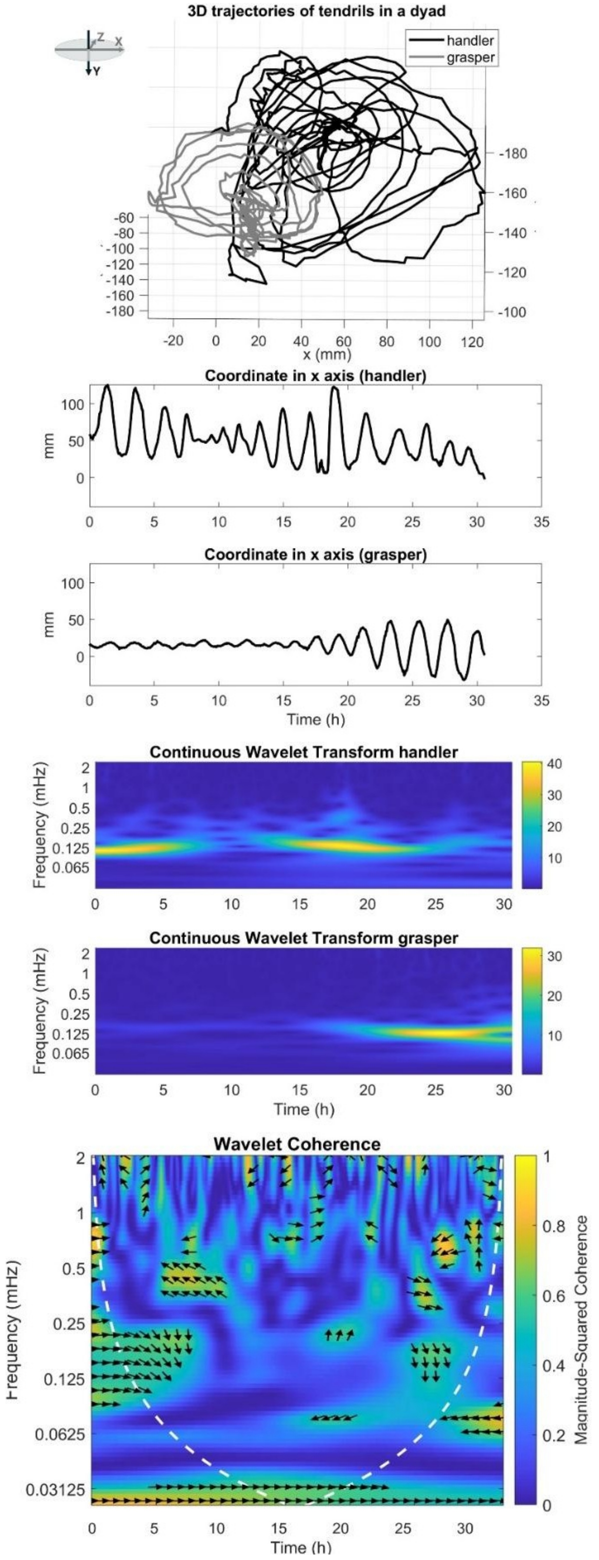

Figure S3. A) 3D trajectories of the tendril of both handler and grasper for a sample real dyad. Y axis represent the vertical axis while X and Z axes are on the horizontal plane; B) Time series of the x coordinate for the tendrils of the handler and the grasper plant corresponding to the 3D trajectory in A); C) continuous wavelet transform (CWT) of the time series of x coordinate presented in B); D) WTC matrix obtained from the CWTs in C). In the WTC matrix, the arrows represent the lead-lag phase relationship between the two time series at each time-frequency point. A zero-phase difference, represented by arrows pointing directly to the right, indicates that the series are perfectly in phase at that scale. Conversely, arrows pointing to the left indicate anti-phase (i.e., the series move in opposite directions). Arrows pointing right-down or left-up indicate that the first variable (handler) is leading, while arrows pointing right-up or left-down suggest that the second variable (grasper) is leading. Phase arrows are only displayed in regions where the squared wavelet coherence exceeds the threshold of 0.5. The white dotted lines in WTC matrices indicate the cone of inffuence (COI), where edge effects become significant. Interpretation outside the COI is less reliable due to boundary artifacts.

Additional sample real dyad #4

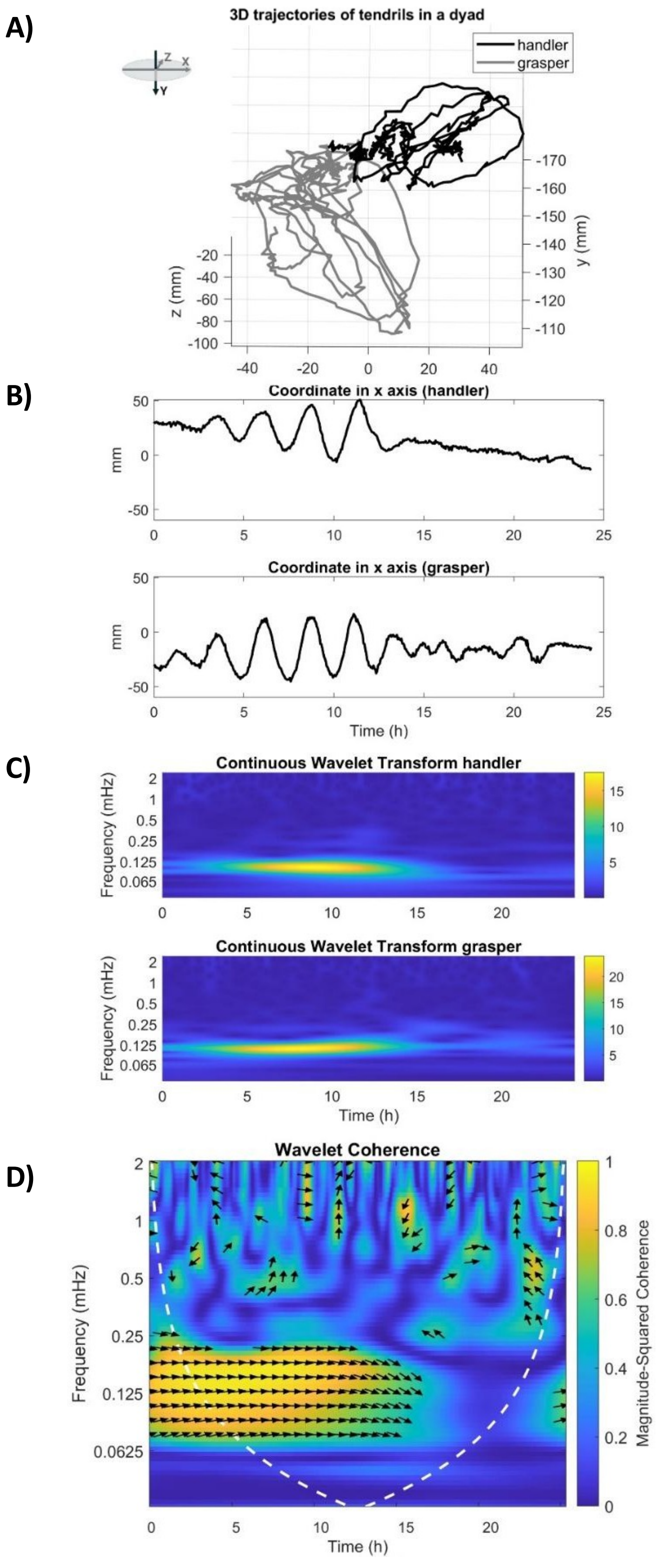

Figure S4. A) 3D trajectories of the tendril of both handler and grasper for a sample real dyad. Y axis represent the vertical axis while X and Z axes are on the horizontal plane; B) Time series of the x coordinate for the tendrils of the handler and the grasper plant corresponding to the 3D trajectory in A); C) continuous wavelet transform (CWT) of the time series of x coordinate presented in B); D) WTC matrix obtained from the CWTs in C). In the WTC matrix, the arrows represent the lead-lag phase relationship between the two time series at each time-frequency point. A zero-phase difference, represented by arrows pointing directly to the right, indicates that the series are perfectly in phase at that scale. Conversely, arrows pointing to the left indicate anti-phase (i.e., the series move in opposite directions). Arrows pointing right-down or left-up indicate that the first variable (handler) is leading, while arrows pointing right-up or left-down suggest that the second variable (grasper) is leading. Phase arrows are only displayed in regions where the squared wavelet coherence exceeds the threshold of 0.5. The white dotted lines in WTC matrices indicate the cone of inffuence (COI), where edge effects become significant. Interpretation outside the COI is less reliable due to boundary artifacts.

Additional sample real dyad #5

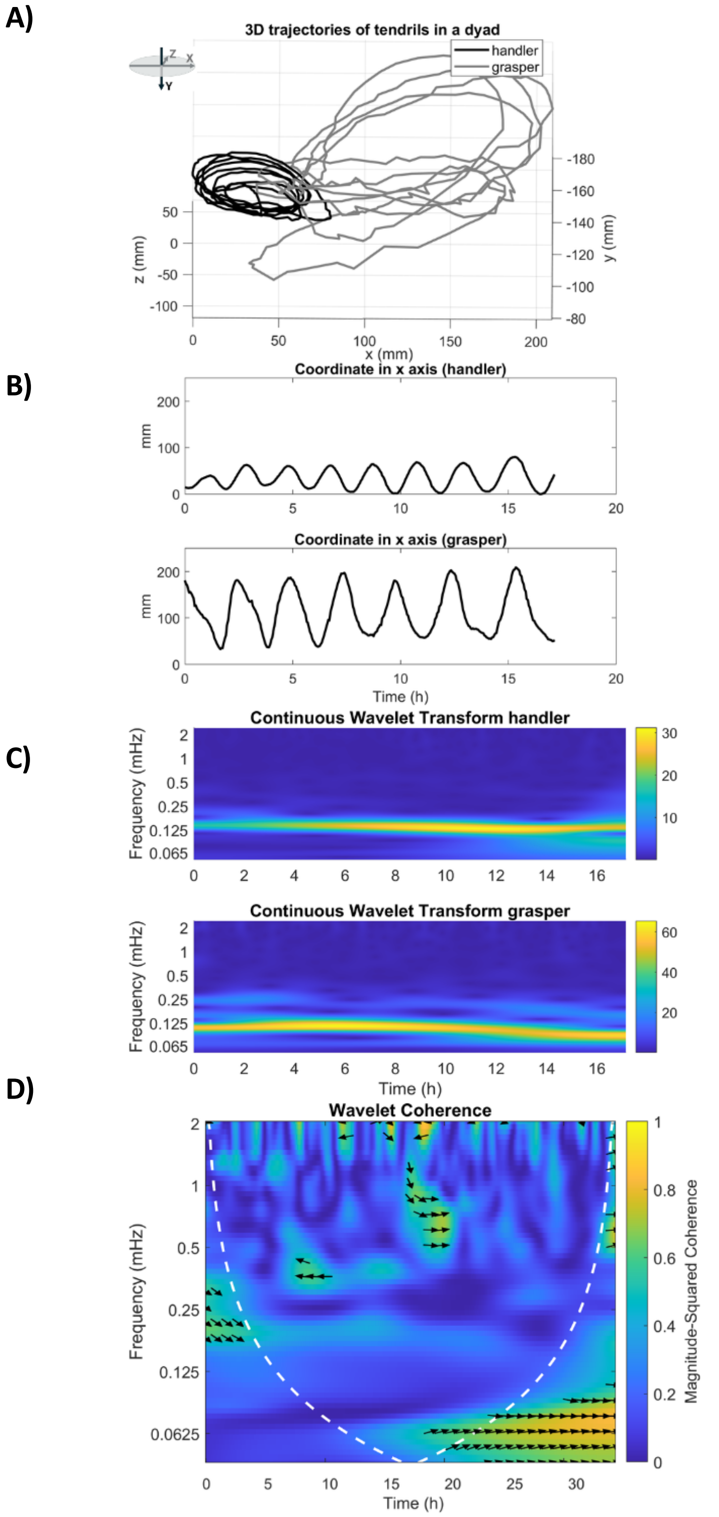

Figure S5. A) 3D trajectories of the tendril of both handler and grasper for a sample real dyad. Y axis represent the vertical axis while X and Z axes are on the horizontal plane; B) Time series of the x coordinate for the tendrils of the handler and the grasper plant corresponding to the 3D trajectory in A); C) continuous wavelet transform (CWT) of the time series of x coordinate presented in B); D) WTC matrix obtained from the CWTs in C). In the WTC matrix, the arrows represent the lead-lag phase relationship between the two time series at each time-frequency point. A zero-phase difference, represented by arrows pointing directly to the right, indicates that the series are perfectly in phase at that scale. Conversely, arrows pointing to the left indicate anti-phase (i.e., the series move in opposite directions). Arrows pointing right-down or left-up indicate that the first variable (handler) is leading, while arrows pointing right-up or left-down suggest that the second variable (grasper) is leading. Phase arrows are only displayed in regions where the squared wavelet coherence exceeds the threshold of 0.5. The white dotted lines in WTC matrices indicate the cone of inffuence (COI), where edge effects become significant. Interpretation outside the COI is less reliable due to boundary artifacts.

Additional sample real dyad #6

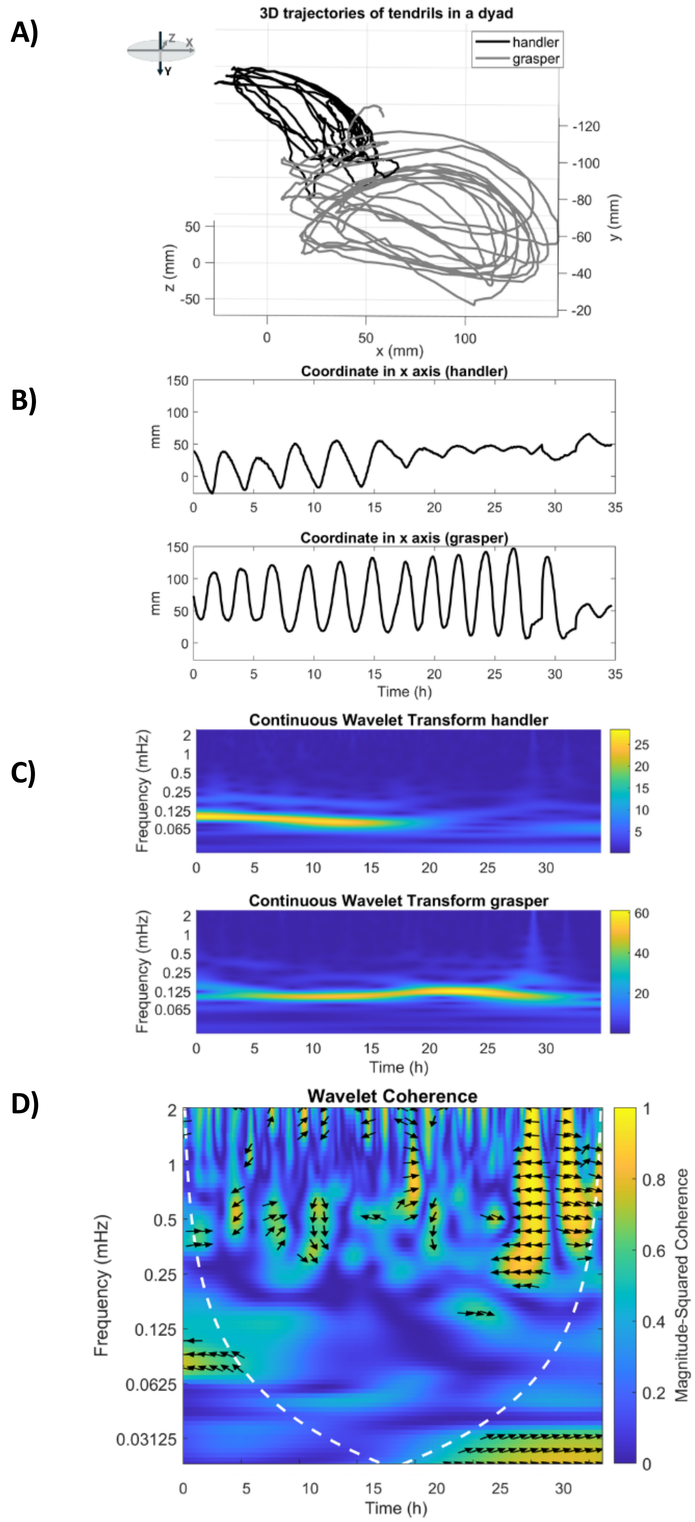

Figure S6. A) 3D trajectories of the tendril of both handler and grasper for a sample real dyad. Y axis represent the vertical axis while X and Z axes are on the horizontal plane; B) Time series of the x coordinate for the tendrils of the handler and the grasper plant corresponding to the 3D trajectory in A); C) continuous wavelet transform (CWT) of the time series of x coordinate presented in B); D) WTC matrix obtained from the CWTs in C). In the WTC matrix, the arrows represent the lead-lag phase relationship between the two time series at each time-frequency point. A zero-phase difference, represented by arrows pointing directly to the right, indicates that the series are perfectly in phase at that scale. Conversely, arrows pointing to the left indicate anti-phase (i.e., the series move in opposite directions). Arrows pointing right-down or left-up indicate that the first variable (handler) is leading, while arrows pointing right-up or left-down suggest that the second variable (grasper) is leading. Phase arrows are only displayed in regions where the squared wavelet coherence exceeds the threshold of 0.5. The white dotted lines in WTC matrices indicate the cone of inffuence (COI), where edge effects become significant. Interpretation outside the COI is less reliable due to boundary artifacts.

Additional sample real dyad #7

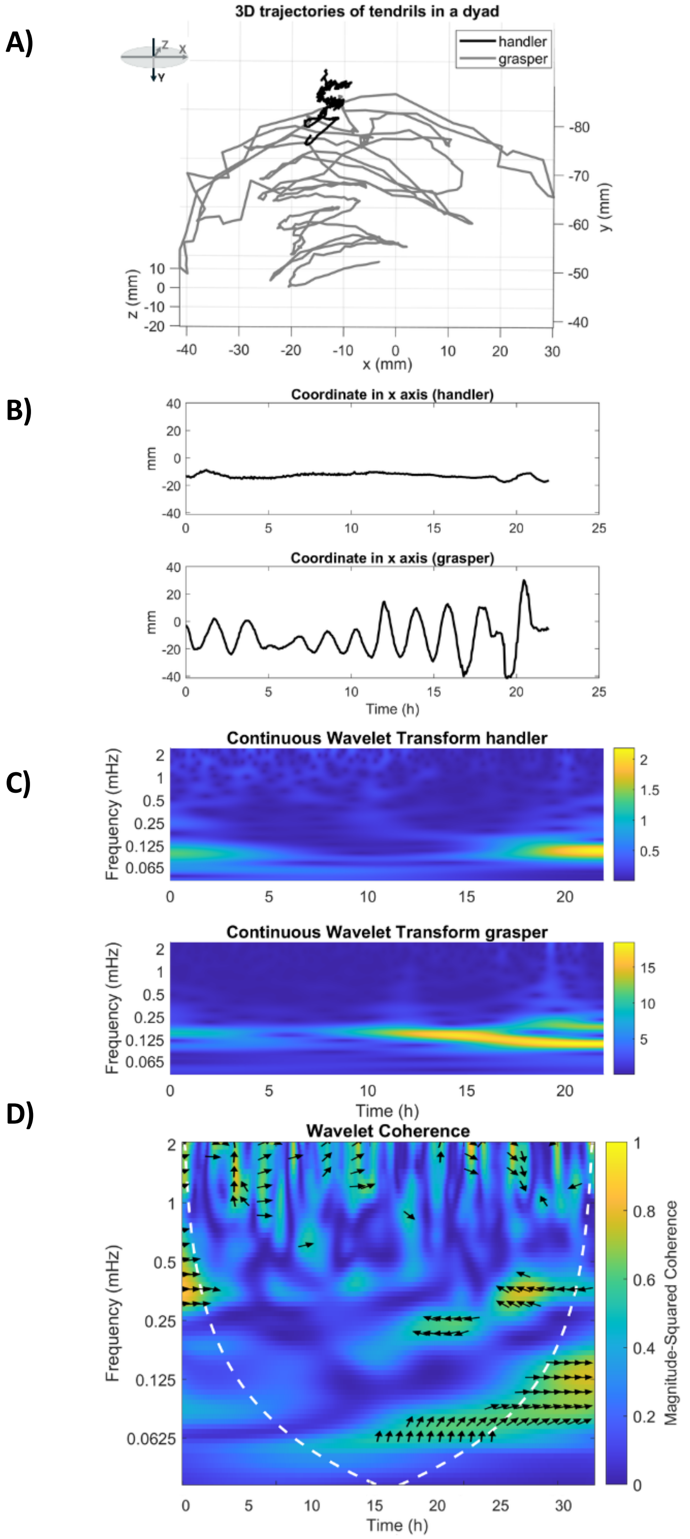

Figure S7. A) 3D trajectories of the tendril of both handler and grasper for a sample real dyad. Y axis represent the vertical axis while X and Z axes are on the horizontal plane; B) Time series of the x coordinate for the tendrils of the handler and the grasper plant corresponding to the 3D trajectory in A); C) continuous wavelet transform (CWT) of the time series of x coordinate presented in B); D) WTC matrix obtained from the CWTs in C). In the WTC matrix, the arrows represent the lead-lag phase relationship between the two time series at each time-frequency point. A zero-phase difference, represented by arrows pointing directly to the right, indicates that the series are perfectly in phase at that scale. Conversely, arrows pointing to the left indicate anti-phase (i.e., the series move in opposite directions). Arrows pointing right-down or left-up indicate that the first variable (handler) is leading, while arrows pointing right-up or left-down suggest that the second variable (grasper) is leading. Phase arrows are only displayed in regions where the squared wavelet coherence exceeds the threshold of 0.5. The white dotted lines in WTC matrices indicate the cone of inffuence (COI), where edge effects become significant. Interpretation outside the COI is less reliable due to boundary artifacts.
